# Therapeutic Potential of Local Application of Fibroblast Growth Factor-2 to Periodontal Defects in a Preclinical Osteoporosis Model

**DOI:** 10.3390/bioengineering12070748

**Published:** 2025-07-09

**Authors:** Shinta Mori, Sho Mano, Naoki Miyata, Tasuku Murakami, Wataru Yoshida, Kentaro Imamura, Atsushi Saito

**Affiliations:** 1 Department of Periodontology, Tokyo Dental College, Chiyoda-ku, Tokyo 1010061, Japan; morishinta@tdc.ac.jp (S.M.); smano@tdc.ac.jp (S.M.); miyatanaoki@tdc.ac.jp (N.M.); murakamitasukutdc@gmail.com (T.M.); yoshidawataru2023@gmail.com (W.Y.); imamurakentarou@tdc.ac.jp (K.I.); 2 Oral Health Science Center, Tokyo Dental College, Chiyoda-ku, Tokyo 1010061, Japan

**Keywords:** osteoporosis, periodontitis, fibroblast growth factor-2, periodontal regeneration

## Abstract

This study investigated the effects of local fibroblast growth factor (FGF)-2 application on periodontal healing in an osteoporotic model, both in vivo and in vitro. Wistar rats were divided into the ovariectomy (OVX) and Control groups. Periodontal defects were created 8 weeks post-OVX and treated with hydroxypropylcellulose (HPC) or FGF-2 + HPC. Healing was evaluated through micro-computed tomography and histological analyses at 2 and 4 weeks. In vitro, bone marrow mesenchymal stromal cells (BMSCs) were cultured with/without FGF-2 and assessed for cell morphology, viability/proliferation, and osteoblastic marker expression. Alkaline phosphatase (ALP) staining was also performed. FGF-2-treated defects in both groups showed significantly greater bone volume fraction, trabecular number, and thickness compared to HPC only. Histologically, FGF-2 enhanced new bone formation, with the greatest levels in the Control group. In vitro, OVX BMSCs showed reduced actin staining versus controls. FGF-2 increased cell viability/proliferation and protrusions in both groups while downregulating *Alpl* and *Bglap* expression levels and reducing ALP-positive cells. FGF-2 increased new bone formation in the OVX group, stimulated proliferation of OVX BMSCs, and modulated their differentiation. FGF-2 could enhance periodontal healing even under osteoporotic conditions, albeit to a lesser extent.

## 1. Introduction

Osteoporosis is one of the most common chronic bone diseases worldwide. The prevalence of osteoporosis in the world is 18.3% [1]. There are approximately 16 million osteoporotic patients in Japan [2]. Osteoporosis develops when bone mineral density and bone mass decrease, or when the structure and strength of the bone change [3]. It is generally caused by an imbalance between osteoclasts and osteoblasts due to estrogen deficiency in postmenopausal women [4].

Periodontitis is caused by bacterial infection, but its outcome and response to treatment are closely related to the host’s systemic condition and environmental factors [5]. Among these conditions, osteoporosis is significantly associated with an increased risk of periodontal disease [6]. Patients with osteoporosis exhibit significantly higher levels of clinical attachment loss [7] and lower bone density and bone mass in the jaw [8] compared to healthy individuals. The relationship between osteoporosis and periodontal health extends to treatment outcomes as well. Bone destruction as a result of osteoporosis can have an adverse effect on the bone repair process [9], including delayed alveolar bone repair after tooth extraction and reduced bone mass after healing [10]. This is consistent with the findings that postmenopausal osteoporotic women experience poorer bone healing after fractures [11]. Consequently, osteoporosis may not only increase the severity of periodontal disease but also interfere with healing after periodontal treatment or contribute to disease recurrence [12].

Periodontal regenerative therapy improves the prognosis of periodontitis [13]. Key components in periodontal tissue engineering include progenitor cells, signaling molecules, scaffolds, and blood supply [14]. One of the signaling molecules, fibroblast growth factor (FGF)-2, promotes cell proliferation and differentiation during periodontal healing and stimulates angiogenic activity [15]. In Japan, a recombinant human FGF-2 formulation has been successfully used for periodontal regeneration since 2016 [16,17]. Diabetes mellitus is also an established risk factor for periodontal bone loss [18]. In vivo studies have shown that diabetes affects alveolar bone remodeling and wound healing [19]. A significant increase in bone volume fraction and trabecular thickness was observed after the local application of FGF-2 to periodontal defects in diabetic rats [20]. In an osteoporotic rat model, the local application of enamel matrix derivative (EMD) improved periodontal healing [21]. In a previous study, the intraosseous injection of FGF-2 increased bone density in osteoporotic rat femurs [22]. However, the effects of FGF-2 on periodontal healing under osteoporotic conditions remain unclear. Understanding how a locally applied growth factor performs in a compromised bone environment should provide important insights into the development of more efficient periodontal regenerative therapies.

This study aimed to investigate the effects of local FGF-2 application on two fronts: the healing of surgically created periodontal defects in an osteoporosis rat model, and the behavior of bone marrow mesenchymal stromal cells (BMSCs) obtained from osteoporotic rats.

## 2. Materials and Methods

### 2.1. Animals

Forty-four female Wistar rats, weighing 200–250 g and aged 10 weeks (Sankyo Labo Service, Tokyo, Japan), were used. They were housed individually under standard laboratory conditions, with unrestricted access to water and standard rat chow. All experimental procedures complied with the Treatment of Experimental Animals protocol of Tokyo Dental College (no. 242203), and this study followed the ARRIVE guidelines.

### 2.2. Ovariectomy (OVX)

The in vivo model of osteoporosis was established by ovariectomy (OVX) in half of the rats, performed under aseptic conditions using a minimally invasive surgical technique as described in previous protocols [23]. General anesthesia was induced by intraperitoneal injection of a combination of medetomidine (0.15 mg/kg), midazolam (2 mg/kg), and butorphanol (2.5 mg/kg). Linear bilateral lumbar incisions of 10 mm were made on the lateral skin. After blunt dissection of the muscle and peritoneum, the bilateral ovaries were excised after ligation of the ovarian artery and vein using absorbable sutures (Vicryl^®^ 6-0; Johnson & Johnson, Tokyo, Japan). The incisions were then closed in layers with nylon 4-0 sutures. Control animals underwent sham surgery.

### 2.3. Confirmation of Osteoporotic Conditions

At 8 weeks post-OVX, the trabecular bone structure of the rat femurs was analyzed to confirm the osteoporotic condition. The femurs were evaluated using a microcomputed tomography (micro-CT) system (R-mCT; Rigaku, Tokyo, Japan). For evaluation, a 2 mm region of interest (ROI) was defined in the rat femur. This ROI was established 1 mm proximal to the epiphyseal border of the growth plate, extending for a length of 2 mm in the proximal direction. It comprised a series of acquired slices. Image data obtained were analyzed using a software package (TRI/3D-BON ver R.10.01.03.15-H-64; Ratoc System Engineering, Tokyo, Japan). After 3D reconstruction, the bone volume fraction (bone volume/total volume: BV/TV), trabecular number (Tb.N), trabecular thickness (Tb.Th), and trabecular separation (Tb.Sp) were determined to identify the osteoporotic condition. The trabecular bone region was extracted as a low bone mineral density (BMD) region surrounded by cortical bone, which indicates a medium BMD region [24]. To evaluate BMD, a calibration curve was constructed using a phantom (Kyoto Kagaku, Kyoto, Japan).

### 2.4. Preparation of Fibroblast Growth Factor-2 (FGF-2)

Recombinant FGF-2 (Lot no.90 HCC) was first diluted in distilled water and subsequently combined with 3% hydroxypropyl cellulose (HPC) (FUJIFILM Wako Pure Chemical, Osaka, Japan) to prepare a 0.3% FGF-2 formulation for in vivo applications.

### 2.5. In Vivo Model and Surgical Interventions

The in vivo experimental model and surgical interventions are shown in Supplemental Figure S1. Periodontal defects were created at 8 weeks following the establishment of osteoporotic conditions. Animals were divided into the following four subgroups: (1) Control/HPC (*n* = 11), (2) Control/FGF-2 (*n* = 11), (3) OVX/HPC (*n* = 11), and (4) OVX/FGF-2 (*n* = 11) (Figure S1a). Anesthesia was induced through the intraperitoneal administration of a combination of medetomidine (0.15 mg/kg), midazolam (2 mg/kg), and butorphanol (2.5 mg/kg). Additionally, local infiltration anesthesia was applied using 2% xylocaine with 1:80,000 adrenaline. After performing a crestal incision and elevating a full-thickness flap, standardized periodontal defects measuring 2.0 mm × 2.0 mm× 1.7 mm (width × length × depth) were created mesial to the maxillary first molars (M1) [24] (Figure S1b–d). This was accomplished using a surgical template [25] (Figure S1e). The surgery was performed under magnification using a ×4.3 loupe with an LED light. The periodontal ligament (PDL) and cementum were carefully removed from the M1 root. The defects were then irrigated with saline and dried with sterile gauze. The defects in each group received FGF-2 with HPC (30 µL) or HPC only (Figure S1f). The flaps were closed with 6-0 absorbable sutures (Figure S1g). For pain control, acetaminophen was given.

### 2.6. Microcomputed Tomography of Surgical Periodontal Defect

At 2 or 4 weeks post-periodontal surgery, the animals were anesthetized, and cardiovascular perfusion was performed with 4% paraformaldehyde solution (FUJIFILM Wako Pure Chemical). The maxillae were harvested, and the healing of the surgical defects was assessed using a micro-CT system (R-mCT; Rigaku) under the exposure conditions of 90 kV and 150 μA. Imaging was performed at ×10 magnification with a slice width of 16 µm. The information from slices was stored at a resolution of 484 × 481 pixels. We defined the ROI by the previously described method [24]. The obtained data were analyzed using a software package (TRI/3D-BON; Ratoc System Engineering). Within the ROI, newly formed bone was defined as the region with BMD (400 to 1000 mg/cm^3^) [26]. The new bone region was obtained by subtracting the defined regions for existing bone from the total radiopaque region with BMD above 400 mg/cm^3^. The analysis focused on BV/TV, Tb.N, Tb.Th, and Tb.Sp within the ROI. The micro-CT data underwent evaluation by a lead examiner blinded to the experimental group assignments, and this was subsequently verified by a second, independent examiner.

### 2.7. Histological Analysis

A sagittal cut was made through the palatal midline to prepare the maxillae for histologic evaluation. After fixation in buffered 4% paraformaldehyde solution for 24 h, the specimens were demineralized in 10% ethylenediaminetetraacetic acid disodium salt (EDTA-2Na, pH 7.0) (FUJIFILM Wako Pure Chemical) at 4 °C for 3 weeks and then embedded in paraffin. Mesial to distal sections of 5 μm thickness were cut with a microtome (Hyrax M25; Carl Zeiss, Oberkochen, Germany). The sections obtained from each specimen, especially those corresponding to the central part of the root within the defect, were stained with hematoxylin–eosin.

### 2.8. In Vitro Cell Culture

Rat bone marrow mesenchymal stromal cells (BMSCs) from the Control and OVX groups were isolated and cultured according to the previously reported method with minor modifications [27]. Briefly, the previous study used Dulbecco’s modified eagle medium, while we used α-minimal essential medium (αMEM, Gibco, Invitrogen, Carlsbad, CA, USA) containing 10% heat-inactivated fetal bovine serum and antimicrobials. At 8 weeks following OVX or sham surgery, femurs of each rat were dissected under sterile conditions. All connective tissues and muscles were detached. After removal of the epiphysis, bone marrow from the bilateral rat femoral diaphyses were washed with culture medium through a 21-gauge needle inserted into the shaft of the bone. Bone marrow cells were incubated at 37 °C in 5% CO_2_ in air. The medium was changed every 2–3 days. When the cells approached 80% confluence, adherent cells were treated with 0.25% trypsin solution and passaged. BMSCs are characterized by their ability to adhere to plastic surfaces under standard culture conditions and by their typical spindle-shaped and fibroblast-like morphology [28]. Since the cells at passages 2–4 showed these characteristics, we identified them as BMSCs and used them for subsequent experiments.

### 2.9. Assessment of Cell Morphology

The morphology of BMSCs was evaluated by phase-contrast microscopy and confocal laser scanning microscopy (CLSM). Images were visualized using an all-in-one microscope (BZ-X800, KEYENCE, Osaka, Japan) with a phase-contrast objective (×100).

For CLSM, at 24 h post-seeding, the samples were fixed in 4% paraformaldehyde for 20 min, followed by a 60 min incubation with 10% goat serum (FUJIFILM Wako Pure Chemical) and a 5 min permeabilization using 0.1% Triton X-100 at room temperature. Alexa Fluor-488 Phalloidin (1:100) (Life Technologies, Carlsbad, CA, USA) was used to label actin, and nuclei were counterstained with 4′,6-diamidino-2-phenylindole (DAPI) (1:1000 dilution) (Thermo Scientific, Pittsburgh, PA, USA). Imaging was conducted using a CLSM (LSM880, Carl Zeiss) with a 10× objective (eyepiece magnification; 10×). Z-stack images were acquired with 2 μm intervals, utilizing excitation wavelengths of 405 and 488 nm, and maximum projections of the stacks were processed via ZEN 2 black (ver 14.0.18.201; Carl Zeiss).

### 2.10. Assessment of Cell Viability/Proliferation

To evaluate the effects of FGF-2 on cell viability/proliferation, BMSCs (at passages 2–4) were incubated in 24-well plates (1 × 10^4^ cells/well), with/without 100 µL of FGF-2-containing culture medium. At various timepoints, the WST-8 assay (Cell Counting Kit-8; Dojindo Laboratories, Kumamoto, Japan) was conducted in accordance with the manufacturer’s protocol.

### 2.11. Quantitative Reverse Transcription-PCR (qRT-PCR)

The expression levels of *Alpl* and *Bglap* in BMSCs were analyzed by qRT-PCR at 3, 7, and 14 days of incubation. Total RNA was extracted from cells cultured with or without FGF-2 using the RNeasy^®^ Mini Kit (Qiagen, Valencia, CA, USA). Quantitative real-time PCR (qRT-PCR) analysis was conducted using the 7500 Fast Real-Time PCR system (Thermo Fisher Scientific, Waltham, MA, USA). The primer sequences used are shown in Supplemental file Table S1. GAPDH was measured as an internal control, and the relative expression levels were calculated using the 2^−∆∆Ct^ method.

### 2.12. Alkaline Phosphatase (ALP) Staining

To detect alkaline phosphatase (ALP) activities in BMSCs, ALP staining was performed. BMSCs were seeded (1 × 10^4^ cells/well) in 24-well plates with/without 100 µL of FGF-2-containing culture medium. At 7 days, the staining was performed using an ALP staining kit (FUJIFILM Wako Pure Chemical) following the manufacturer’s protocol. Images were visualized using an all-in-one microscope (BZ-X800, KEYENCE, Osaka) with a bright field objective (×20).

The ALP-positive area was quantified using image processing software (ImageJ ver.1.54p; https://imagej.net/ij/). Four fields (540 µm × 720 µm) were randomly selected from each well, and their average values were calculated to represent the data for that well. The proportion of the ALP-positive area was then compared between groups. In this study, the ALP-positive area was used to assess osteoblast activity, as differentiated osteoblasts express ALP over larger areas, which more effectively reflects bone formation potential.

### 2.13. Statistical Analysis

The sample size was calculated through a power analysis, ensuring a 90% power at a 0.05 two-sided significance level. This calculation was based on a 10.5% difference in bone volume between groups, as assessed by micro-CT, and a standard deviation of 7% [24]. Based on this determination, a sample size of 10 (sites) was required for each group at each time point. Taking into account the 10% dropout, the sample size was set at *n* = 11.

Intergroup comparisons for micro-CT analysis were made by two-way analysis of variance (ANOVA) with Tukey’s post hoc test. For the WST-8 assay, intragroup comparisons were evaluated by the Kruskal–Wallis test with Dunn’s post hoc test. Intergroup differences for the WST-8 assay were evaluated by the Mann–Whitney U test. Intergroup comparisons for qRT-PCR analyses were made by two-way ANOVA with Tukey’s post hoc test. A software package (Prism ver 10.4; GraphPad, Boston, MA, USA) was used. A *p*-value < 0.05 was deemed significant.

## 3. Results

### 3.1. Establishment of Osteoporotic Model

The establishment of OVX-induced osteoporosis was confirmed by micro-CT analysis, with reference to the following characteristics: reductions in BV/TV (approximately 40%), Tb.N (30%), and Tb.Th (20%) and an increase in Tb.Sp (50%) [29]. The 3D micro-CT images of the femurs of OVX rats showed a striking decrease in the subchondral trabecular bone density, disrupted microstructure, and enlarged marrow cavities compared to the Control animals (Figure S2a–d). Our analysis showed significant reductions in BV/TV, Tb.N, and Tb.Th (by approximately 60%, 51%, and 13%, respectively) and an increase in Tb.Sp (by approximately 205%) (Figure S2e–h), confirming the establishment of osteoporotic conditions.

### 3.2. Micro-CT Analysis of Periodontal Defects and Their Healing

**Figure 1:**
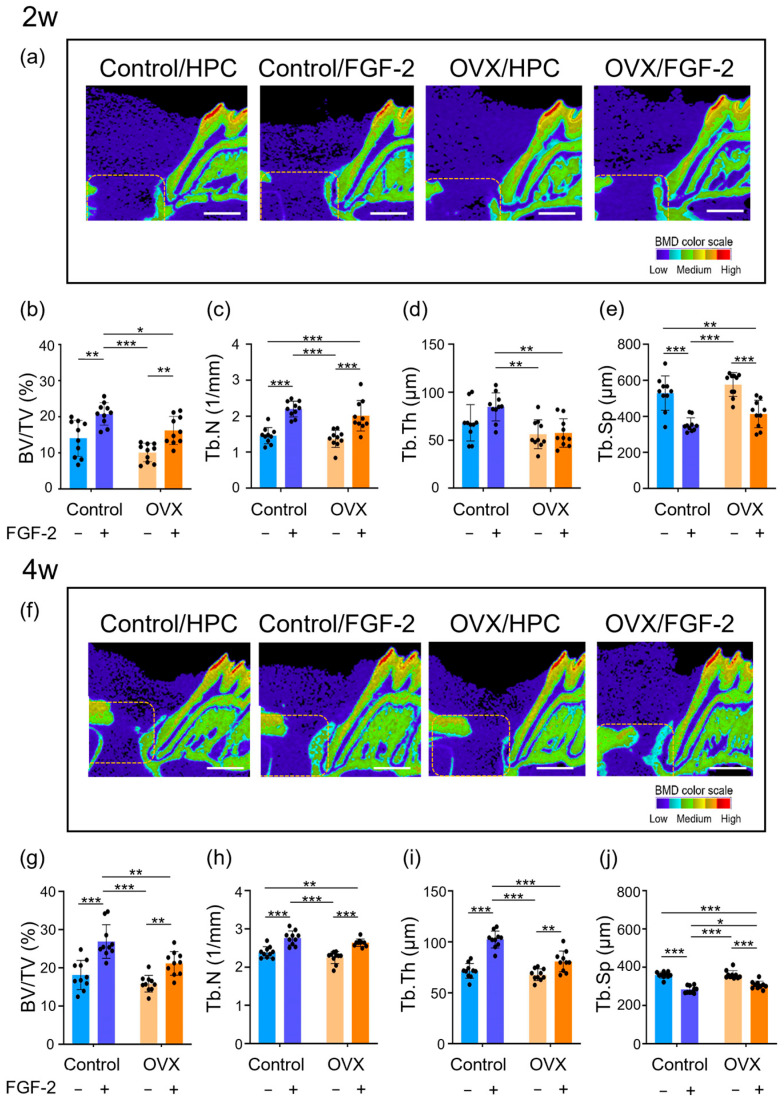
Sagittal micro-CT images at 2 and 4 weeks and their quantification. The color scale indicates the following: red and orange = high bone mineral density (BMD), yellow and green = medium, and light blue and dark blue = low. (**a**,**f**) Dotted line indicates the surgical defect margin; bar = 1000 µm. (**b**–**e**,**g**–**j**) Quantitative analysis by 3-D structural analysis software. Bone volume/total volume (BV/TV) (**b**,**g**), trabecular number (Tb.N) (**c**,**h**), trabecular thickness (Tb.Th) (**d**,**i**), and trabecular separation (Tb.Sp) (**e**,**j**) were compared between groups. Data are presented as mean ± SD (*n* = 10). * *p* < 0.05, ** *p* < 0.01, *** *p* < 0.001 by two-way ANOVA with Tukey’s post-test.

One rat in each group died within the first 2 weeks postoperatively, and they were excluded from the following analyses. The results from micro-CT analysis are presented in Figure 1. Sagittal slice images from micro-CT obtained at 2 weeks post-periodontal surgery showed restricted bone formation in the HPC groups, and low BMD structures were observed (Figure 1a). In both the OVX and Control groups, no discernible adverse effects were noted during the first 2-week observation period following the local application of FGF-2. Novel bone was identified close to the root in the FGF-2-applied groups. Figure 1b–e shows the quantitative analysis of newly formed bone after 2 weeks. Treatment of defects with FGF-2 significantly increased BV/TV and Tb.N in both the OVX and Control groups (Figure 1b,c). FGF-2 treatment yielded no significant change in Tb.Th in both the Control and OVX groups (Figure 1d). In contrast, Tb.Sp was significantly reduced in the FGF-2-treated groups (Figure 1e).

After 4 weeks, new bone formation was limited in the OVX/HPC group, whereas it was increased in the other groups (Figure 1f). In the Control groups, BMD values of newly formed bone fell within the medium range (green), while the OVX/FGF-2 group exhibited values in the low to medium range (light blue), indicating reduced mineralization. FGF-2 treatment significantly increased BV/TV, Tb.N, and Tb.Th while decreasing Tb.Sp in both the OVX and Control groups (Figure 1g–j). FGF-2 yielded significantly higher BV/TV and Tb.Th values in the Control group than in the OVX group (Figure 1g,i).

### 3.3. Histological Findings

**Figure 2:**
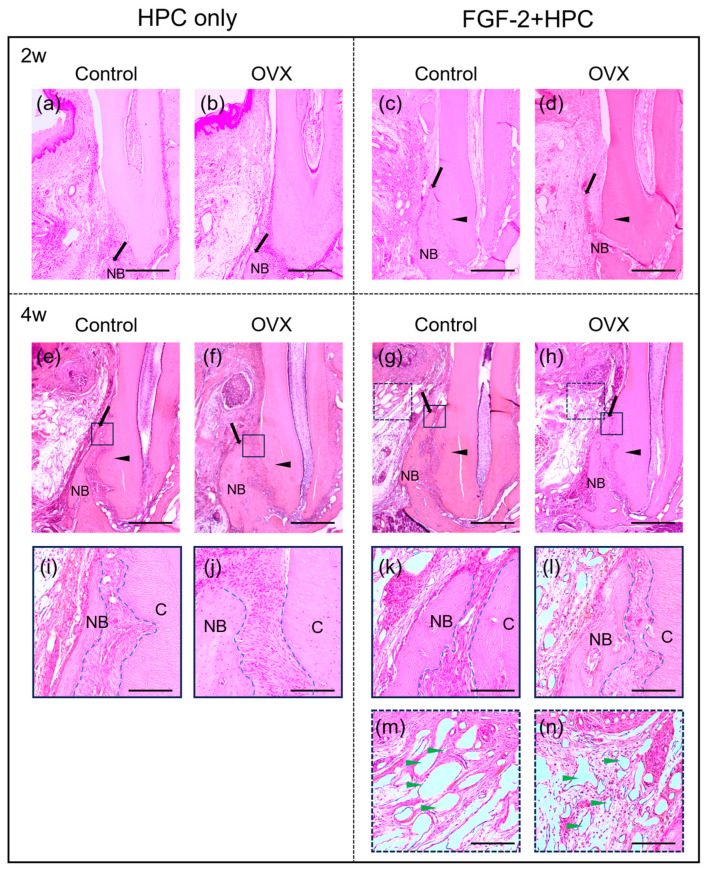
Histological analysis (H&E staining). (**a**–**d**) Photomicrographs of histological sections from periodontal defects adjacent to the root surface at 2 weeks postoperatively (original magnification ×25; bar = 500 µm; black arrowheads indicate new cementum; black arrows indicate the most coronal extent of new bone (NB)). (**a**,**b**) The HPC only group shows limited new bone formation. (**c**,**d**) In the FGF-2-treated groups, newly formed bone can be observed from the root side of the previous defects. (**e**–**h**) Images at 4 weeks postoperatively (original magnification ×25; bar = 500 µm). (**f**) The OVX/HPC group shows limited new bone formation. (**e**,**g**,**h**) The levels of new bone in the Control/HPC, Control/FGF-2, and OVX/FGF-2 groups appear to be greater than the OVX/HPC group. (**i**–**l**) Higher magnification images of the solid framed area within the corresponding group at 4 weeks (original magnification ×200; scale bar = 100 µm; C, cementum). Periodontal ligament-like tissue is observed between the root surface and the newly formed bone (the region between dotted lines). (**m**,**n**) High magnification of the dotted framed area within the corresponding group at 4 weeks (original magnification ×100; bar = 200 µm). Vascular structures (green arrowheads) can be observed in connective tissues.

The histological findings are illustrated in Figure 2. At 2 weeks postoperatively, there was no clear difference in new bone formation between the Control/HPC group (Figure 2a) and the OVX/HPC group (Figure 2b). The previous defects were filled with connective tissue. Although there was no obvious difference in the coronal extent of new bone between the Control/FGF-2 (Figure 2c) and OVX/FGF-2 (Figure 2d) groups, the thickness of the newly formed bone appeared to be reduced in the OVX/FGF-2 group. The formation of PDL-like tissue was observed between the novel bone and root surface, and no signs of ankylosis were observed (Figure 2c,d). The HPC groups (Figure 2a,b) presented limited new bone compared with the FGF-2 groups (Figure 2c,d). In the FGF-2 groups (Figure 2c,d), new bone formation was detected originating from the root aspect of the previous defects.

At 4 weeks postoperatively, compared with the Control/HPC group (Figure 2e), the OVX/HPC group (Figure 2f) showed lower levels of new bone. Luminal structures within the connective tissue were observed more frequently in the Control/HPC group (Figure 2e) than in the OVX/HPC group (Figure 2f). Compared with the Control/FGF-2 group (Figure 2g), the level of newly formed bone was lower in the OVX/FGF-2 group (Figure 2h). Between the FGF-2 groups, no obvious structural differences in bone were observed (Figure 2g,h). FGF-2 administration (Figure 2g,h) yielded greater levels of new bone formation compared with the HPC only groups (Figure 2e,f). Compared with the other groups (Figure 2e,f,h), the level of novel bone appeared to be greater in the Control/FGF-2 group (Figure 2g). In all groups (Figure 2e–h), the dentin surface exposed by root planing was covered by new cementum. There was no ankylosis, and PDL-like tissue was observed between the root surface and newly formed bone (Figure 2i–l). In the FGF-2 groups, vascular-like structures were prevalent (Figure 2g,h). Cells with oval nuclei were lining the lumen in a single row (Figure 2m,n).

### 3.4. In Vitro Cell Morphology

**Figure 3:**
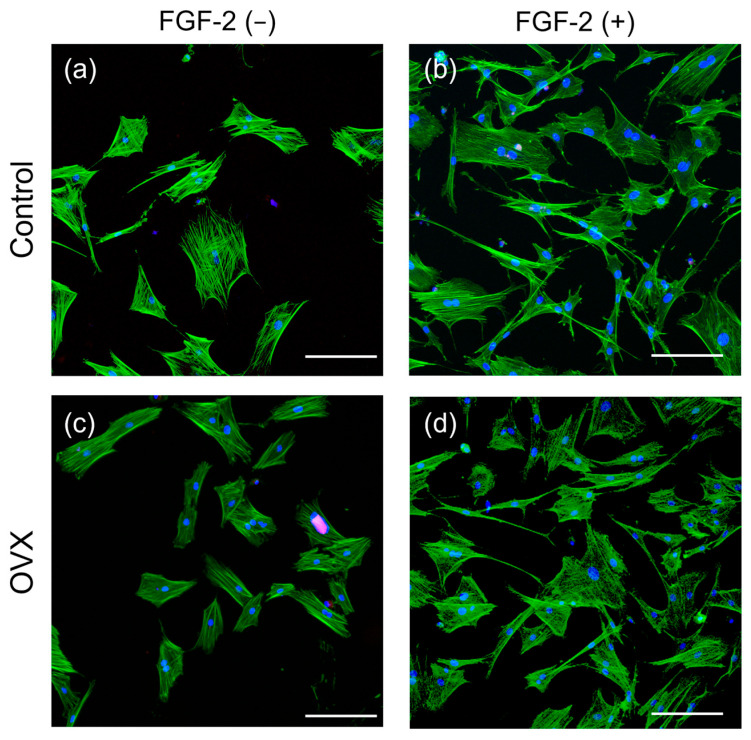
CLSM images of bone marrow mesenchymal stromal cells (BMSCs). BMSCs were cultured with/without FGF-2 for 24 h and analyzed by CLSM. (**a**) Unsupplemented Control (non-OVX) cells, (**b**) FGF-2-supplemented Control cells, (**c**) unsupplemented OVX cells, (**d**) FGF-2-supplemented OVX cells. A series of Z-stack images was acquired with 2-μm intervals, utilizing excitation wavelengths of 405 and 488 nm, and maximum projections of the stacks were processed via ZEN 2 black software. The cells are labelled for actin (green) and the nucleus (blue). The purple spot in (**c**) is an artifact. (bar = 200 μm).

In phase-contrast microscopy, BMSCs were observed adhering to the bottom of the dish and presented a spindle-shaped morphology at 24 h after seeding (Supplemental file Figure S3). CLSM images of the cells after 24 h of culture are shown in Figure 3. When compared with Control BMSCs (Figure 3a), OVX BMSCs (Figure 3c) appeared to have lower staining intensity in the actin cytoskeleton. Compared with the unsupplemented cells (Figure 3a,c), a greater number of elongated cells attached to the dish bottom when cultured with FGF-2 (Figure 3b,d). Cell protrusions were more apparent. Furthermore, the bundles of actin filaments in the FGF-2 groups (Figure 3b,d) were more prominent than in the unsupplemented groups (Figure 3a,c). With FGF-2 supplementation (Figure 3b,d), the cells were elongated, and no substantial difference in the morphology was observed between the Control and OVX groups.

### 3.5. Cell Viability/Proliferation

**Figure 4:**
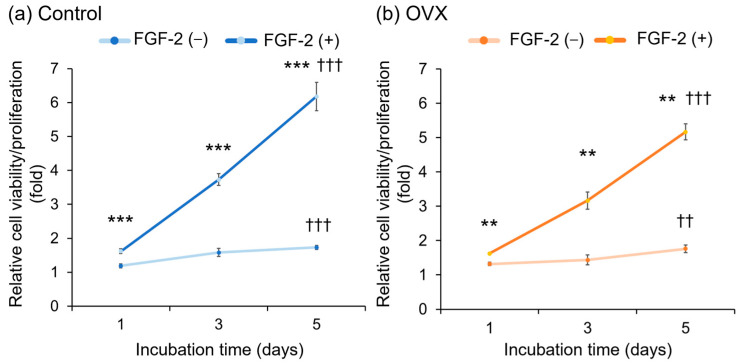
Viability/proliferation of bone marrow mesenchymal stromal cells (BMSCs). BMSCs (at passages 2–4) were seeded with/without FGF-2 and allowed to grow for up to 5 days. (**a**) Control (non-OVX) cells. (**b**) OVX cells. The WST-8 assay was employed to assess cell viability and proliferation. Data are shown as mean ± SD (*n* = 6). ** *p* < 0.01, *** *p* < 0.001 significant difference between groups by the Mann–Whitney U test. ^††^ *p* < 0.01, ^†††^ *p* < 0.001 significant difference from values at 1 day by Kruskal–Wallis test with Dunn’s post-test.

The results of the cell viability/proliferation assays are shown in Figure 4. A significant increase in the values was observed at 5 days of incubation compared with 1 day in all groups. There were no obvious differences in the values between the Control group (Figure 4a) and the OVX group (Figure 4b) without FGF-2. At each time point, the FGF-2-supplemented groups yielded significantly greater values than the unsupplemented groups.

### 3.6. Expression of Osteoblastic Differentiation Markers

**Figure 5:**
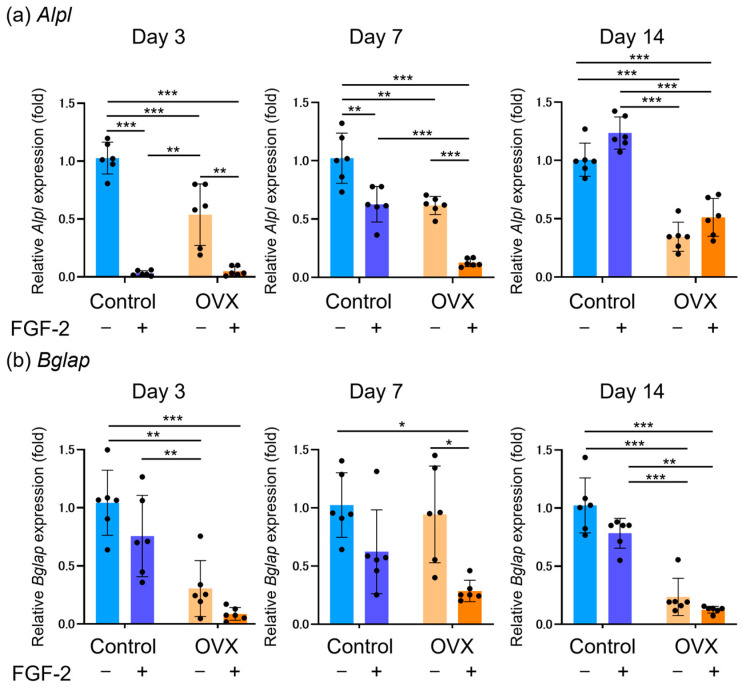
Expression levels of osteoblastic differentiation markers in bone marrow mesenchymal stromal cells (BMSCs). Relative expression levels of *Alpl* (**a**) and *Bglap* (**b**) incubated with/without FGF-2 are shown. Values are expressed relative to the Control (non-OVX) group. Data are shown as mean ± SD (*n* = 6). * *p* < 0.05, ** *p* < 0.01, *** *p* < 0.001 by two-way ANOVA with Tukey’s post-test. *Alpl*, alkaline phosphatase; *Bglap*, osteocalcin.

The expression levels of *Alpl* and *Bglap* in the BMSCs at various time points are shown in Figure 5. *Alpl* expression in the OVX BMSCs was significantly reduced compared with the Control (non-OVX) cells (Figure 5a). In both the Control and OVX cells, FGF-2 treatment significantly reduced *Alpl* expression on day 3 and day 7, but the reduction was no longer observed on day 14.

*Bglap* expression in the OVX BMSCs was significantly lower than in the Control cells on days 3 and 14 (Figure 5b). Treatment with FGF-2 significantly reduced *Bglap* expression only in the OVX BMSCs on day 7.

### 3.7. Assessment of ALP Staining

**Figure 6:**
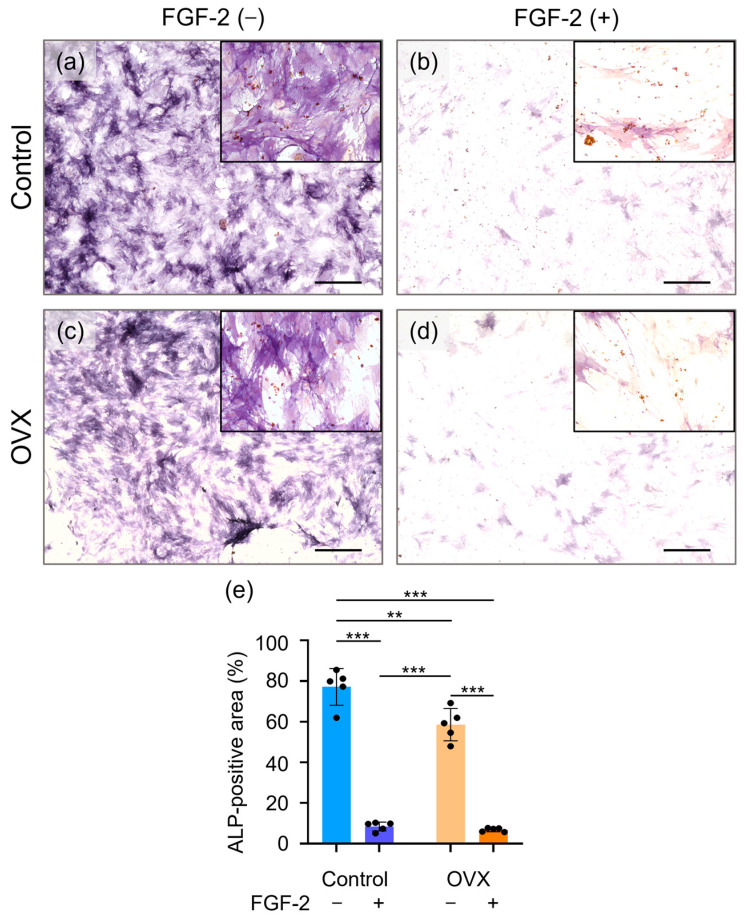
Representative photomicrographs of ALP staining of BMSCs incubated for 7 days. (**a**) Control (non-OVX) cells, (**b**) FGF-2-supplemented Control cells, (**c**) OVX cells, and (**d**) FGF-2-supplemented OVX cells (original magnification ×4; bar = 500 µm). Higher-magnification images (×20) are shown in the insets. (**e**) Quantification of the proportion of ALP-positive area, by using ImageJ. Data are shown as mean ± SD (*n* = 5). ** *p* < 0.01, *** *p* < 0.001 by two-way ANOVA with Tukey’s post-test.

The results of ALP staining of BMSCs are shown in Figure 6. After 7 days of seeding, when compared with the Control group (Figure 6a), the prevalence of ALP-positive cells appeared to be reduced in the OVX group (Figure 6c). FGF-2 supplementation exhibited a trend toward reducing the ALP-positive area in the Control (Figure 6b) and OVX (Figure 6d) groups.

OVX BMSCs showed a significantly reduced ALP-positive area compared with the Control cells (Figure 6e). The ALP-positive area was significantly decreased by FGF-2 supplementation.

## 4. Discussion

The present study investigated the effects of local FGF-2 application on periodontal healing in osteoporotic rats. Our results demonstrated enhanced new bone formation following FGF-2 treatment even under osteoporotic conditions, with FGF-2 stimulating BMSC proliferation and modulating cell differentiation in vitro. These findings may have important implications for treating periodontitis in patients with osteoporosis and other compromised bone conditions.

In osteoporotic patients, bone modeling and remodeling become unbalanced. As a result, they lose bone mass, and their bone microarchitecture deteriorates. Reduced improvement following periodontal therapy has been noted in osteoporotic patients compared with those who received osteoporosis treatment [30]. In the present study, there was no significant difference in BV/TV between the Control and OVX groups that did not receive FGF-2. In the FGF-2-treated groups, the OVX group presented a significantly lower BV/TV than the non-OVX (Control) group at 2 and 4 weeks after periodontal surgery. However, in the OVX group, treatment with FGF-2 significantly increased BV/TV. In patients with osteoporosis, the mechanisms of bone metabolism are disrupted, impairing bone repair and regeneration [31]. Our findings support the notion that osteoporosis can complicate the treatment of periodontitis due to the disruption in bone metabolism [32]. It is also suggested that the regenerative effect of FGF-2 can be expected even under osteoporotic conditions.

The micro-CT analysis showed that FGF-2 treatment resulted in significantly higher BV/TV values and Tb.N than with HPC only in both the OVX and Control groups at 2 and 4 weeks postoperatively and significantly increased Tb.Th at 4 weeks. In contrast, the FGF-2 treatment significantly reduced Tb.Sp at 2 and 4 weeks postoperatively. However, even with FGF-2 treatment, the OVX group showed significantly lower BV/TV values and trabecular thickness and significantly wider trabecular separation when compared with the FGF-2-treated Control group. In the histological analysis at 4 weeks postoperatively, the FGF-2-treated groups showed a greater extent of newly formed bone compared with the untreated groups at 4 weeks. The new bone formation in the OVX/FGF-2 group appeared to be inferior to that in the Control/FGF-2 group. A study using a similar osteoporotic rat model reported that the use of EMD yielded a significant increase in new bone formation in periodontal defects [20]. However, the healing was significantly less in OVX animals than in controls. A previous study found that the local administration of FGF-2 to fracture sites in rabbit tibiae resulted in significantly greater trabecular bone mass compared with the vehicle control at 4 weeks post-administration [33]. In another study, the local application of FGF-2 to periodontal defects in dogs was shown to significantly increase new bone formation [34]. In addition, a previous in vitro study reported that FGF-2 induced the expression of vascular endothelial growth factor and promoted angiogenesis [35]. This is particularly important given the reduced angiogenesis in osteoporosis [36]. Our histological observation revealed more pronounced vascular-like structures in the FGF-2-treated groups. Therefore, it is suggested that FGF-2 acts as a factor that promotes angiogenesis even in osteoporotic conditions. Moreover, the addition of FGF-2 promoted the coronal extension of the new bone even in the OVX group. One of the notable findings of the micro-CT analysis was that FGF-2 treatment improved the BV/TV and Tb.Th values in the osteoporotic rats, but the levels of improvement were inferior compared with the FGF-2-treated Control group. This may indicate that although FGF-2 treatment improves bone quality, its effectiveness is limited under osteoporotic conditions.

The reduced proliferative activity and differentiation ability of BMSCs in osteoporotic conditions result in impaired bone repair [37]. In a previous study, BMSCs derived from OVX mice showed impaired osteogenic differentiation [38]. In addition, osteoporosis enhances periodontal tissue destruction by reducing the proliferative capacity of osteoblasts and activating the bone-resorptive activity of osteoclasts [37]. Suppression of MSCs’ proliferation, osteoblastic differentiation, and osteogenic potential in the early stages of healing has been suggested as a mechanism underlying poor bone formation in osteoporosis [39]. These changes likely contribute to the reduced repair capacity observed in osteoporotic models. In the present study, when compared with BMSCs from the Control group, the OVX group showed significantly lower expression of *Alpl* (up to 14 days of incubation) and *Bglap* (on day 3 and day 14). In the ALP staining at 7 days, the prevalence of ALP-positive cells was significantly reduced in the OVX group compared with the Control group. In a study using BMSCs from normal and osteoporotic rats, the level of ALP staining following 7 days of osteogenic induction was lower in the OVX BMSCs compared with Sham BMSCs [40]. Reduced osteogenic differentiation, as observed in the OVX BMSCs in the present study, is consistent with previous evidence that osteoporotic conditions impair the functions of BMSCs [40,41]. In addition, the suppressed cell viability/proliferation rates over time in unsupplemented OVX BMSCs further highlight the adverse effects of osteoporosis. This reduced cellular activity is likely to play a role in impaired tissue regeneration, as the proliferative capacity of stem cells is critical in the early stages of healing. Furthermore, regarding the cytoskeleton, actin filament dynamics are essential for cell migration, adhesion, and differentiation, and their disruption could impair the ability to contribute effectively to bone repair [42]. Estrogen deficiency has been shown to inhibit the formation of actin fibers in an osteocyte-like cell line [43]. The low intensity of actin filaments observed in the OVX BMSCs may also contribute to reduced migratory capacity, impaired adhesion and reduced osteogenic differentiation, resulting in impaired bone repair.

In our in vitro experiments, FGF-2 increased the number of BMSCs showing an elongated morphology. Cells cultured with FGF-2 demonstrated significantly greater viability and proliferation compared to those cultured without it. FGF-2 has been shown to enhance cell proliferation by upregulating the expression of CD44, a molecule associated with cell adhesion and proliferation [44]. These results suggest that FGF-2 promoted the initial adhesion to and spreading of adherent cells on culture dishes and contributes to the promotion of cell proliferation.

In a canine periodontal defect, FGF-2 has been demonstrated to enhance cell proliferation and stimulate the expression of osteoblastic differentiation markers within one week post-surgery [45]. In the present study, FGF-2 suppressed the expressions of *Alpl* (at 3 and 7 days), and *Bglap* (at 7 days) in BMSCs from both the OVX and Control groups. Our in vitro data indicated that the addition of FGF-2 promoted cell proliferation but suppressed differentiation during the early stage. Because the OVX BMSCs may possess an inherently lower differentiation potential compared to the Control cells, the downregulation of the *Alpl* and *Bglap* expression levels in the OVX BMSCs may not be the sole effect of FGF-2. Furthermore, in the present study, ALP staining of the OVX BMSCs revealed that FGF-2 significantly reduced ALP-positive cells following 7 days of incubation. These data suggest that FGF-2 inhibits the differentiation of BMSCs toward major bone cell lineages during the early phase of incubation. It should be noted that inherent heterogeneity exists in primary BMSC populations, which naturally contain subpopulations at various stages of lineage commitment. The heterogeneous nature of BMSCs is well-documented in the literature, with primary isolates typically containing a mixture of truly multipotent stem cells, lineage-primed progenitors with varying degrees of commitment, and cells that have already begun spontaneous differentiation toward specific lineages. Given this context, our results should be interpreted as follows: 1. The baseline expression levels in our control groups represent the endogenous osteogenic potential of our BMSC populations rather than a truly undifferentiated state. 2. The FGF-2 treatment effects should be viewed as an enhancement of existing osteogenic tendencies rather than de novo induction of differentiation. 3. The magnitude of change, rather than the absolute values, provides the most meaningful comparison between treatment groups. It has been reported that, in the early stages of periodontal healing, FGF-2 promoted the proliferation of PDL cells while inhibiting differentiation; however, in the late stages, it stimulated their differentiation into osteoblasts [15]. The in vitro and in vivo findings from the present study demonstrated the disruption of bone repair in osteoporosis while also revealing that FGF-2 still promotes novel bone formation, albeit with attenuated yet substantial efficacy compared to non-osteoporotic states. These results collectively suggest that even in osteoporotic condition, FGF-2 contributes to periodontal healing by promoting the proliferation of undifferentiated mesenchymal cells within the defect and regulating their differentiation into osteoblasts.

There are limitations in the present study. The potential risk related to the use of the growth factor was not investigated in this article; in addition to the therapeutic effects, potential risks in drug application must be considered. Our in vitro cell populations may not represent classical BMSCs, but rather, a mixed population with significant lineage bias. While this limitation affects the interpretation of our results, the biological phenomena we observed—regulation of osteogenic differentiation in response to FGF-2 treatment—remain valid within this context. The clinical relevance may actually be enhanced, as these lineage-committed cells may be more representative of the cell populations that would be therapeutically relevant in periodontal regeneration applications. Our experimental model does not consider the influence of bacteria on periodontal defect formation, which may limit its direct relevance to human periodontitis. While the general bone metabolism induced by OVX in rats is considered analogous to postmenopausal osteoporosis in humans [46,47], the current data reflect relatively short-term effects of osteoporotic conditions on periodontal tissues. Given the high prevalence of osteoporosis in humans, future studies using appropriate osteoporosis models with longer observation periods may provide deeper insights into the effects of FGF-2 on periodontal regeneration under compromised bone conditions.

## 5. Conclusions

The locally applied FGF-2 enhanced periodontal healing within the defects in both the OVX and non-OVX groups, showing the regenerative effects of FGF-2. In addition, the in vitro data indicated that FGF-2 supplementation not only stimulated the proliferation of BMSCs but also modulated their differentiation into osteogenic lineages. Although its effect may be limited, these results suggest that FGF-2 promotes periodontal regeneration even under conditions of impaired bone metabolism, such as osteoporosis.

## Data Availability

The data that support the findings of this study are available from the corresponding author upon reasonable request.

## Supplementary Materials

The following supporting information can be downloaded at: https://www.mdpi.com/article/10.3390/bioengineering12070748/s1, Figure S1: In vivo experimental protocol and representative images of surgical interventions (FGF-2 group); Figure S2: Micro-CT analysis of rat femurs at 2 months after ovariectomy (OVX) or sham operation; Figure S3: Phase-contrast microscopy of bone marrow mesenchymal stromal cells (BMSCs). Table S1: The primers used for qRT-PCR.

## Abbreviations

The following abbreviations are used in this manuscript:

FGF-2	Fibroblast growth factor-2
OVX	Ovariectomy
PDL	Periodontal ligament
HPC	Hydroxypropylcellulose
ALP	Alkaline phosphatase
BMSCs	Bone marrow mesenchymal stromal cells
CLSM	Confocal laser scanning microscopy
