# Supplementary material for: Therapeutic Potential of Local Application of Fibroblast Growth Factor-2 to Periodontal Defects in a Preclinical Osteoporosis Model
Source: Bioengineering (Basel). 2025 Jul 9;12(7):748. doi: 10.3390/bioengineering12070748 (PMC12292976; doi:10.3390/bioengineering12070748)
Supplement: Supplementary file 1 [file bioengineering-12-00748-s001.zip › bioengineering-3716515-supplementary.pdf]

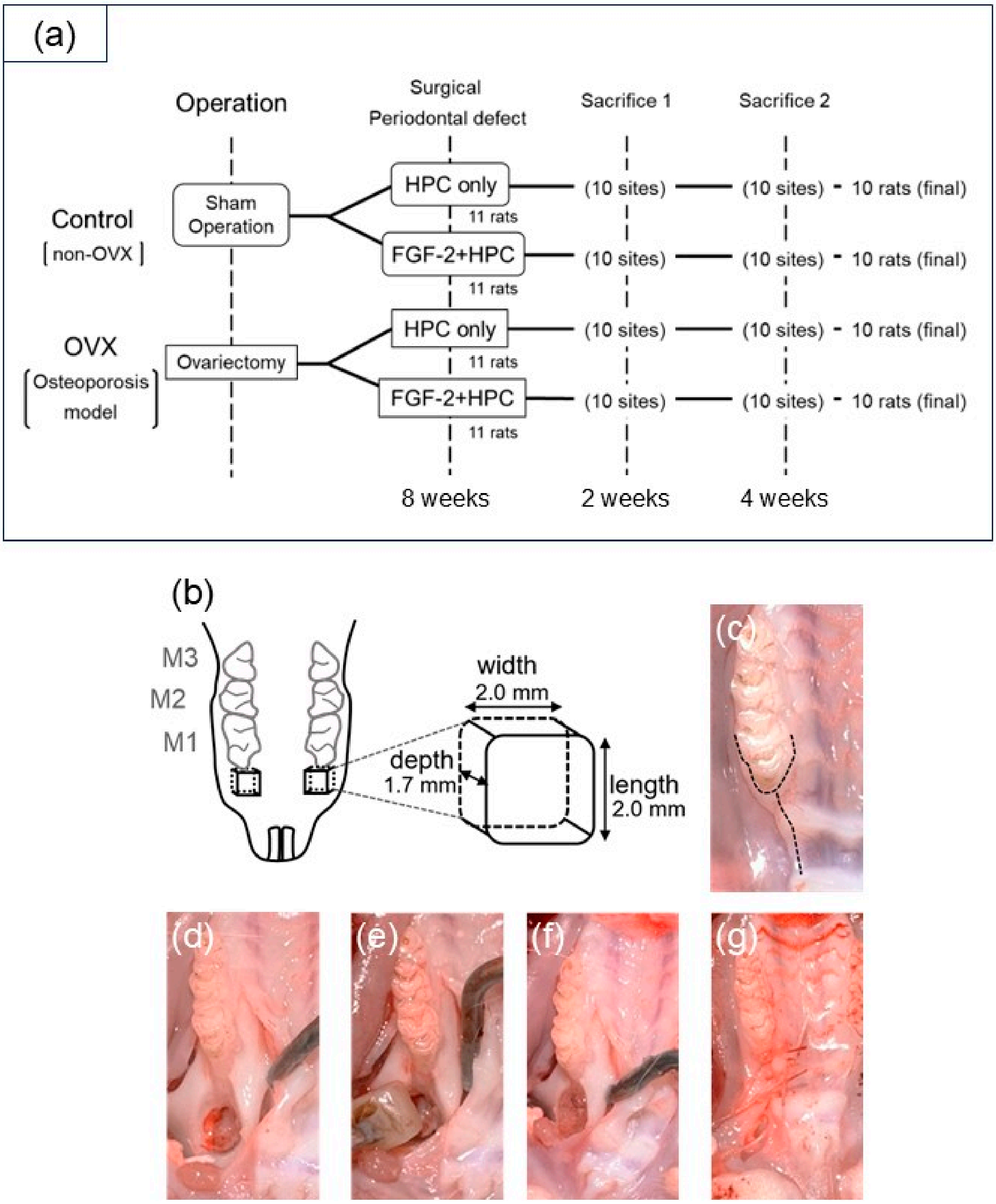

Figure S1. In vivo experimental protocol and representative images of surgical interventions (FGF-2 group).

(a) Experimental protocol and groups (b) Standardized defect size (c) Incision design: black dotted line (d) After raising full-thickness flaps, bilateral standardized periodontal defects (2.0 × 2.0 × 1.7 mm) were created mesially of the maxillary first molars (M1). (e) Use of the surgical template. (f) Application of the mixture of FGF-2 and HPC. (g) Flaps were closed using resorbable sutures.

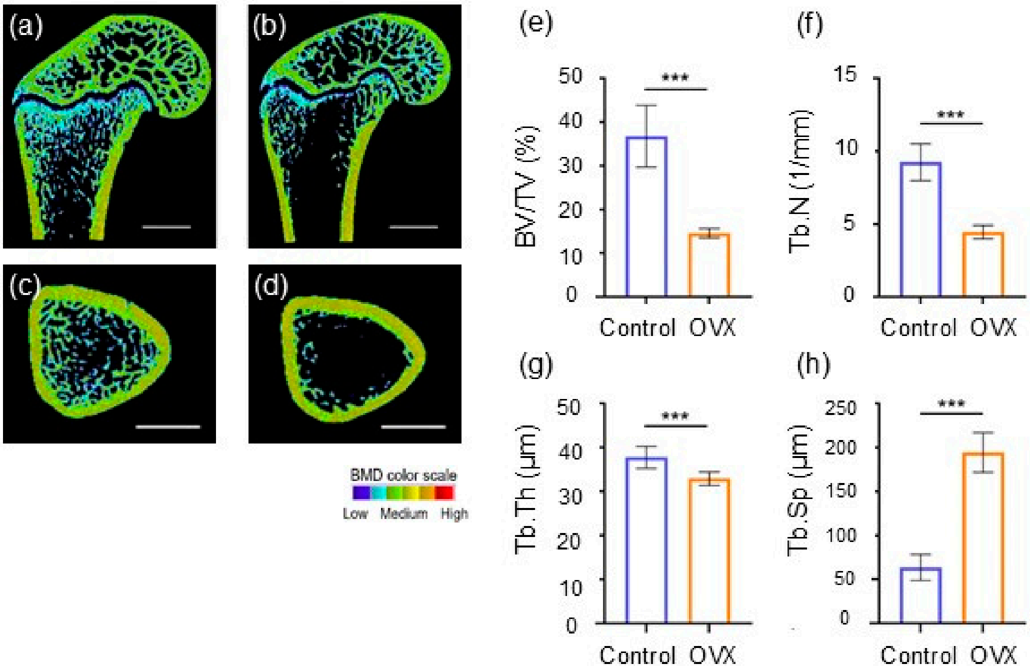

Figure S2. Micro-CT analysis of rat femurs at 8 weeks after ovariectomy (OVX) or sham operation. The color scale indicates: red and orange = high bone mineral density (BMD), yellow and green = medium BMD, and light blue and purple = low BMD. Micro-CT images of the femurs; (a) axial view and (c) transverse view of Control group, (b) axial view and (d) transverse view of OVX group (bar = 1000 µm). Quantitative analysis by the 3-D structural analysis software; (e) Bone volume/Total volume (BV/TV), (f) trabecular number (Tb.N), (g) trabecular thickness (Tb.Th), and (h) trabecular separation (Tb.Sp) were compared between groups. Data shown as mean ± SD (n = 7) *** $p < 0.001$ by Unpaired T test.

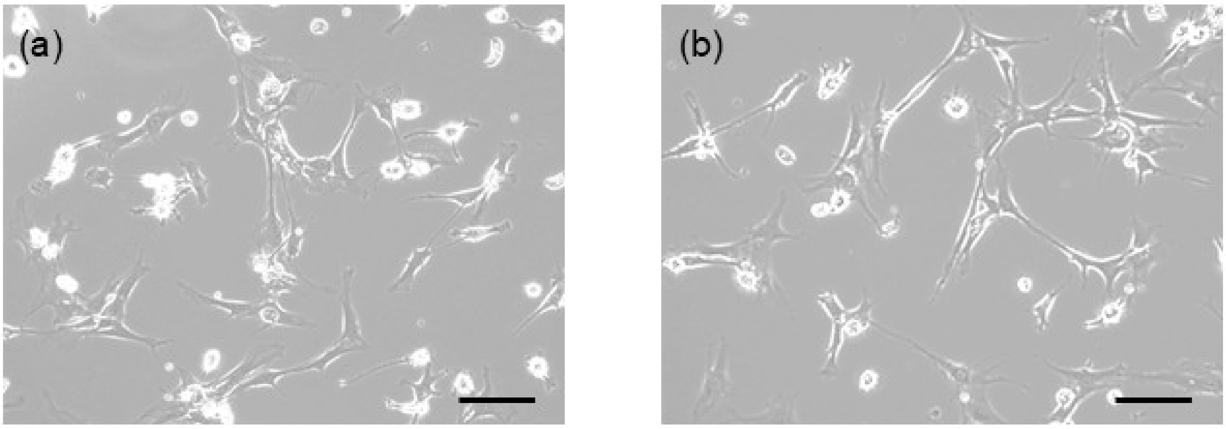

Figure S3. Phase-contrast microscopy of bone marrow mesenchymal stem cells (BMSCs). BMSCs were cultured and observed at 24 hrs after seeding. Images of the Control cells (a) and OVX cells (b) are shown. (Original magnification ×100; bar = 200 μm)

Table S1. The primers used for qRT-PCR

Gene symbol	Gen Bank^{TM} accession no.	Forward primer sequence	Reverse primer sequence
GAPDH	NM_017008.4	TATGACTCTACCCACGGCAA	ATACTCAGCACCAGCATCACC
Alpl	NM_013059.1	AACGTGGCCAAGAACATCATCA	TGTCCATCTCCAGCCGTGTC
Bglap	NM_013414	AAAGCCCAGCGACTCT	CTAAACGGTGGTGCCATAGAT

Alpl, alkaline phosphatase; *Bglap*, osteocalcin.
